# Parents' experiences of having a child who had a stroke: A systematic review and meta‐ethnography

**DOI:** 10.1111/dmcn.70004

**Published:** 2025-09-26

**Authors:** Radhika Bhatia, Shereena Yoon, Ellie Simpson, Nancy McStravick, Vuokko Wallace

**Affiliations:** ^1^ Department of Psychology University of Bath Bath UK

## Abstract

**Aim:**

To explore and synthesize interview‐based, qualitative research of parents' experiences of having a child who had a paediatric stroke.

**Method:**

A systematic search of literature identified seven studies that used qualitative, interview‐based methods to investigate parents' experiences of having a child (younger than 25 years) who had a paediatric stroke. Data were extracted and appraised for quality. All seven studies were included in a meta‐ethnography. An advisory group of two parents contributed to the analysis through two consultation meetings.

**Results:**

A conceptual model of parents' experiences was developed, highlighting the lack of childhood stroke awareness, insufficient support from healthcare professionals, and the role of societal norms. These factors significantly shaped both the emotional impact on parents and the trajectory of their experiences, from emergency response to adjustment.

**Interpretation:**

Greater awareness of childhood stroke, alongside sustained professional support for families, may substantially improve outcomes for parents and their children.

## Graphical abstract

Key elements of parents' experiences consisted of three parts: influencing factors, trajectory, and impact. Unawareness of childhood stroke and insufficient healthcare support were key influencing factors highlighted by parents, and normative ideals were found to be particularly influential. The trajectory reflects parents' different stages of experiences and perspectives following stroke. This is mapped over time and across levels of society. Given the evolving nature of developmental conditions, parents may cycle through the stages of the trajectory as new challenges emerge over time.

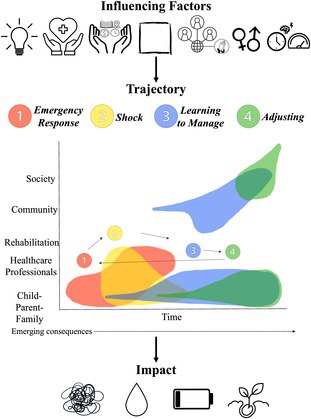

Plain language summary: https://onlinelibrary.wiley.com/doi/10.1111/dmcn.70071

**Abbreviation**
CASP: Critical Appraisal Skills Programme

**What this paper adds**

- Awareness of childhood stroke is lacking in public and medical communities.
- Unawareness intensifies parents' emotional distress following their child's stroke.
- Parents perceive healthcare support to be insufficient.
- Parents experience a trajectory from shock to adjustment.
- Parents strive for normality but can expand this definition over time.

Stroke is globally recognized as a leading cause of disability, with increasing prevalence in recent years.^1^ Since 2009, national campaigns in the UK have aimed to raise awareness of the signs and symptoms of stroke, with recent efforts encouraging immediate action.^2^ The psychological consequences of stroke are increasingly recognized, with individuals who survive it being more likely to experience depression than the general population.^3^, ^4^ The aetiology of post‐stroke depression is multifactorial, involving previous mental health history, stroke severity, age under 70 years, with social participation offering protective benefits.^5^ These findings have informed UK guidance recommending greater provision of psychological support.^3^ However, most literature has focused on adult populations, while paediatric stroke—although rare, with estimated prevalence rates ranging from 0.0013% to 0.013% in high‐income countries^6^—remains under‐researched, contributing to delayed identification and a lack of inclusion for improved support provision.

Parenting or taking on the role of primary caregiver (hereafter encompassed in the term ‘parent’), specifically the parent–child relationship, is vital for child development^7^ and naturally involves stresses as changes transpire.^8^, ^9^ Parental stress can adversely affect child adjustment and these effects can be bi‐directional.^10^ When paediatric disability is introduced, families experience significant disruptions, with impacts on parental stress and quality of life, particularly for mothers.^11^, ^12^ These effects are further influenced by socioeconomic factors and condition‐related challenges such as behavioural difficulties. Parents of children with disabilities must continue to provide a nurturing and nourishing environment for their child while taking on new, often complex caregiving roles.^13^, ^14^ They frequently require more social, informational, and emotional support.^15^ One study involving parents of children with neurodisabilities highlights unmet needs related to holistic family support and the impact of this gap on parental mental health.^16^ Social support, in particular, has been identified as a key buffer against distress.^11^ The Lifespan Neurodisability Navigation‐Building Integrated Framework,^17^ based on Bronfenbrenner's ecological systems theory,^18^ emphasizes the importance of surrounding the child and the family with interconnected layers of support to optimize outcomes.

Contextual influences, such as cultural beliefs and dominant social narratives, shape family experiences, including societal views on parenting and disability. Chronosystems (i.e. transitory socio‐historical contexts) bisect every level of the Lifespan Neurodisability Navigation‐Building Integrated Framework,^17^ reflecting changes of perceptions. Currently, research has predominately focused on mothers, often using their accounts to represent both parents' experiences. However, emerging studies suggest fathers may adopt different perspectives, often focusing more broadly on overall family well‐being,^19^ highlighting the need for more inclusive research that captures diverse parental viewpoints. Further, disability was historically associated with shame and exclusion due to moral interpretations, later pathologized through the medical model, and more recently reframed by the social model, which recognizes disabling barriers as socially constructed.^20^ These changing societal views affect how parents perceive disability and are perceived. Parents often report feeling stigmatized because of their child's condition,^21^ with these perceptions negatively affecting their emotional well‐being.^22^ While this remains unexplored in paediatric stroke, research in adult stroke reveals themes of social exclusion and rejection, which contribute to the development of psychosocial difficulties.^23^

In research on paediatric stroke, a systematic review identified parental concerns about their child's mental health and unmet needs for support and information.^24^ Additional studies have highlighted the toll of stroke on family well‐being, finances, and relationships,^25^ and the lack of professional focus on psychosocial consequences.^26^ There is also emerging recognition of the role that parenting characteristics may play in shaping child recovery, including executive functioning.^27^ These findings expose a significant gap in both research and practice around parental emotional well‐being and its influence on child outcomes. To date, research has predominately focused on quantitative measures of motor and cognitive outcomes in children; the limited qualitative research has almost exclusively explored parents' experiences, with no available synthesis of these findings. Qualitative meta‐synthesis is becoming increasingly recognized as a valuable method to enhance understanding and inform practice and policy.^28^ Meta‐ethnography can be adopted in the field of health and social sciences, and is considered especially appropriate when aiming to understand lived experiences.^29^ Meta‐ethnography was chosen for this research for its value in deepening understanding of complex health‐related phenomena^30^ and because the process can lead to the production of novel insights and conceptual developments.^31^

This study, therefore, aimed to explore and synthesize interview‐based, qualitative research of parents' experiences of having a child who had a paediatric stroke, offering a deeper understanding of their perspectives and implications for clinical practice. The research aimed to consolidate knowledge and propose a conceptual model to guide future care and research.

## METHOD

### Research question

This study was guided by a research question developed using the sample, phenomenon of interest, design, evaluation, research type (SPIDER) framework for qualitative systematic reviews^32^ (Table 1): what are the parents' or primary caregivers' experiences of having a child who has had a stroke, as explored through qualitative or mixed‐method interview‐based studies?

**TABLE 1:** Development of research question in line with the SPIDER framework.

SPIDER framework element	Description of element	Application
Sample	What is the target population to be studied?	Parents or primary caregivers
Phenomenon of interest	What is the topic being explored?	Having a child who has had a stroke
Design	What design/methods have been used?	Interviews
Evaluation	What is the assessed outcome?	Experiences
Research type	What kind of research has been conducted?	Qualitative/mixed‐method studies

Abbreviation: SPIDER, sample, phenomenon of interest, design, evaluation, research type.

### Design

A meta‐ethnographic approach was used, following established guidance,^33^ and reported in accordance with eMERge and the Preferred Reporting Items for Systematic Reviews and Meta‐Analyses (PRISMA) reporting guidance.^34^, ^35^ The quality of included studies was assessed using the Critical Appraisal Skills Programme (CASP).^36^ Participatory action principles were also integrated, in line with recommended practice.^37^

### Research team

The research was led by RB, who had a childhood stroke and who has been motivated to offer improvements to this field, drawing on personal experiences during analysis. Supervision was provided by VW who has conducted qualitative research into her own rare childhood condition, oesophageal atresia. ES and SY joined through the University of Bath's Research Apprentice Scheme, contributing to the initial stages of the review, from the search strategy to the quality appraisal. Both brought personal experiences and professional experiences relevant to the topic, alongside a shared interest developing their qualitative research skills. An advisory group of two White mothers from the United Kingdom, each with a child who had a stroke, was consulted twice during data analysis. Their lived experiences were used to deepen the interpretation of findings.

### Search strategy and screening process

The study was pre‐registered on Prospero (identification number CRD42023451755). A comprehensive search strategy (Table 2) was developed by RB with support from ES, SY, and a university subject librarian, and was approved by VW. Systematic searches were conducted in PubMed, Embase (excluding MEDLINE records), PsycInfo, and Scopus. Google Scholar and reference lists of relevant studies were also examined. Searches were conducted by RB and cross‐checked by either SY or ES within 10 days to ensure accuracy. Records were exported into Covidence (Veritas Health Innovation, Australia)^38^ on 23rd February and 24th February 2024, and managed through this software. Searches were re‐run on 5th April 2025, identifying 157 additional studies, none of which met the inclusion criteria. Two reviewers were required for screen and full‐text review. Double independent screening was conducted by ES (98.24%), SY (76.20%), and RB (25.56%), with RB resolving any conflicts. Full‐text review was conducted by ES (50%) or SY (50%) and RB (100%).

**TABLE 2:** Database search terms according to components of the research question corresponding to the SPIDER framework.

Research question according to SPIDER framework elements	Related search terms
Parents or primary caregivers	parent* OR caregiver OR care‐giver OR mother* OR father* OR guardian AND
Having a child …	neonat* OR neo‐pat* perinat* OR peri‐pat* OR prenat* OR pre‐pat* OR postnat* OR post‐nat* OR *infant OR newborn OR child* OR paediatric OR pediatric OR teen* OR adolescen* OR puberty OR pubescent OR youth AND
… who has had a stroke	stroke OR cerebrovascular OR infarct OR hemiparesis OR ‘brain attack’ OR Apoplex* OR ischemi* OR vascular OR embolism OR thrombosis OR seizure OR ictus AND
Interviews	interview OR qualitative AND
Experiences	experien* OR perception OR attitud* OR view * OR feeling OR emotion* OR ‘everyday life’ OR ‘daily life’ OR ‘living with’ OR impact

Abbreviation: SPIDER, sample, phenomenon of interest, design, evaluation, research type.

### Inclusion and exclusion criteria

Inclusion consisted of studies (including those translated from languages other than English) exploring parents' experiences of having a child who had a paediatric stroke using qualitative, interview‐based methods. Studies with mixed methods could be included focusing only on the interview component for quality appraisal and meta‐ethnography. Thirteen excluded studies did not focus on paediatric stroke.^39^, ^40^, ^41^, ^42^, ^43^, ^44^, ^45^, ^46^, ^47^, ^48^, ^49^, ^50^, ^51^ Three studies had the wrong design,^26^, ^52^, ^53^ seven focused on child outcome perceptions,^54^, ^55^, ^56^, ^57^, ^58^, ^59^, ^60^ and one only addressed intervention experiences.^61^ Three studies without full‐text access were also excluded.^62^, ^63^, ^64^ An unpublished thesis^65^ was included, following an adjustment to the inclusion criteria made before synthesis, as it provided unique insights into a subgroup not otherwise represented in the peer‐reviewed literature: fathers. Additionally, a study in which the age of the child's stroke was ambiguous, and in which participants' children were as old as 24 years at the time of interviews,^66^ was also included. This was justified as participants' children were younger than 25 years, a period during which the brain undergoes significant maturation.^67^, ^68^ Furthermore, this was the only study conducted in a low‐ and middle‐income country or in the Global South, which also addressed the experiences of participants whose children had comorbidities. These factors strengthened the rationale for its inclusion, allowing for this meta‐ethnography to encapsulate a broader range of experiences. Decisions were agreed by all members of the research team.

### Quality appraisal

RB, SY, and ES independently completed CASP checklists^36^ for each included study, then met to discuss and reach consensus or consult with VW as needed. Average interrater agreement across all items was 90.37%. Responses and justifications were documented for each CASP item, following the method of another study.^69^ RB later re‐reviewed all studies to ensure all relevant information was collected.

### Data synthesis

Included studies were read in detail in multiple iterations. SY and ES helped develop a summary table in Microsoft Excel, which was reviewed throughout synthesis by RB, and key contextual information was presented to the advisory group. Advisory group members read half the studies each (excluding the unpublished thesis owing to its length) noting key ideas, contrasting views, and any surprises. Partial reading was mitigated by RB reading the studies in full in addition to summaries of all findings provided by ES and SY, ensuring no key themes were missed or overemphasized. A consultation meeting with RB and advisory group members was held to consider findings and interpretations together. Triangulation revealed strong consistency of identified recurring themes. Recurring concepts from the advisory group were colour‐coded and incorporated into synthesis stages.

First‐order and second‐order constructs, along with initial coding concepts, were extracted by RB into Microsoft Excel sheets for each study. These were then compared across studies, grouped by descriptive labels, and re‐read in order of relevance (on the basis of quality and specificity), starting with general studies and ending with the thesis. Particular attention was paid to second‐order constructs during translation (Table S1). Reciprocal and refutational syntheses were created in Microsoft Excel and then ordered chronologically to support a lines‐of‐argument synthesis. Concepts and labels were refined through discussion with the advisory group, leading to the development of third‐order constructs. The input of advisory group members largely confirmed the interpretive framework, but the group also offered meaningful refinements. Third‐order constructs were then used to code each paper in NVivo (Lumivero, Denver, CO, USA),^70^ which was used primarily for verification and structuring, rather than for generating or further abstracting the constructs. Once third‐order constructs had been developed through manual synthesis and consultation with the advisory group, the use of NVivo ensured no relevant data were overlooked. It also supported quote retrieval and thematic structuring during write‐up by making it easier to sort excerpts by construct.

### Assessing the strength of the body of evidence

Each finding from the synthesis was evaluated using the Grading of Recommendations, Assessment, Development and Evaluation (GRADE) Confidence in the Evidence from Reviews of Qualitative Research (CERQual) approach.^71^ This considered methodological limitations, coherence, adequacy, and relevance. Results from this assessment can be found in Table S2.

## RESULTS

Of the 34 studies assessed for eligibility, seven were selected for inclusion in the review and meta‐ethnography (Figure S1). Data were extracted into a summary table (Table S3), including each study's context (author, location, and year), aims, participant selection, design, data collection and analysis methods, and sample details (including available contextual information). The studies were conducted between 2014 and 2022 across five countries: Brazil (*n* = 1),^66^ France (*n* = 1),^72^ the UK (*n* = 2),^73^, ^74^ Switzerland (*n* = 1),^75^ and the USA (*n* = 2).^65^, ^76^ One study was originally written in Portuguese with a version translated into English available on the host website.^66^ Children's ages ranged from 1 to 24 years, and time since stroke onset, which was named for all but one study,^66^ ranged from 9 months to 10 years. Recruitment strategies varied. Four studies recruited participants from single sources, including a previous research pool,^74^ a condition‐specific charity,^76^ an association,^66^ and a hospital‐based parent group.^65^ The remaining studies recruited participants from multiple hospital sites.^72^, ^73^, ^75^

Stroke timing and inclusion criteria differed. One study focused on perinatal stroke identified in the first 3 months of their life,^74^ while another examined neonatal atrial ischaemic strokes.^75^ One study excluded perinatal stroke or unrelated cognitive/behavioural disorders.^72^ Two studies reported on comorbidities, with one study exclusively interviewing parents of children with stroke due to sickle cell disease.^66^ Participants were exclusively mothers in two studies,^66^, ^76^ and predominately mothers in three others.^72^, ^74^, ^75^ Only one study exclusively interviewed fathers,^65^ and another did not specify parental sex or role.^73^ Only the two studies from the USA reported on ethnicity,^65^, ^76^ and religion was only noted in the thesis^65^ and in the sole study conducted in the Global South.^66^ Parental educational background was reported in three studies, ranging from minimally educated^66^ to highly educated.^76^

While all studies explored parents' experiences of having a child who had a stroke, one study focused solely on parents' self‐directed learning experiences^76^ and another predominately on parents' experiences of the diagnostic process of neonatal atrial ischaemic strokes^75^ which was the only study to use mixed methods. Additionally, one study aimed to include parents' perceptions of the quality of care and support strategies used;^73^ another study incorporated a focus on parents' psychological well‐being;^74^ similarly, another focused on the challenges experienced and strategies used by parents;^65^ and another incorporated a focus on parents' awareness of non‐physical consequences of stroke during early phases of rehabilitation.^72^

### Quality appraisal

The quality appraisal resulted in a summary table (Table S4) highlighting our categorical response as well as assigned scores based on the CASP checklist^36^ (see Table S5 for details of identified strengths and weakness for each CASP component). Overall, the included studies were deemed to have clear statements of aims and appropriate qualitative methodologies. Research design, recruitment, and data collection were predominately found to be suitable. Ethics seemed to be well‐considered across all studies except one,^76^ which did not report obtaining ethical approval or preserving participants' anonymity. This lack of transparency questions the credibility and trustworthiness of its findings. Further, researcher reflexivity and consideration of the researcher–participant relationship, in addition to the rigour of analysis, were seen to be ambiguous in most studies. Additionally, one study lacked a clear statement of findings;^75^ while it reported using thematic content analysis, the results closely followed the interview structure and were largely descriptive, with minimal synthesis or interpretation.

### Synthesis

The lines of argument synthesis generated seven third‐order constructs: (1) shock of an inexplicable diagnosis; (2) emergency response and experiences of healthcare professionals; (3) impact to parents, families, and the affected child; (4) learning to manage, and a demand on resources; (5) socio‐spiritual resources; (6) living with and beyond stroke: adjusting, and (7) normative ideals, expectations and comparisons. Results for each of these are given below.

#### Shock of a diagnosis exacerbated by low levels of awareness

Across studies, parents experienced the diagnosis of childhood stroke as profoundly shocking, described as ‘brutal’^72^, ^75^ by participants and as ‘a thunderclap in a calm sky’ by researchers.^72^ The unexpectedness coupled with general unawareness that children can have strokes, ‘I did not even know that a stroke was possible in a child!’,^75^ contributed to disbelief, emotional distress, and difficulty processing information. Only one parent noted previous experience of children with disabilities, which lessened the emotional impact of diagnosis and allowed appropriate support to be more quickly implemented. The lack of awareness of childhood stroke extended to educators and healthcare professionals, sometimes resulting in dismissed concerns and delayed diagnoses. This construct highlights how societal ignorance about childhood stroke influences parents' emotional responses and the quality of early care.

#### Emergency response and perceptions of healthcare professionals' response

Even before receiving a diagnosis, parents were often already in a state of emergency, seeking help and requesting investigations when they noticed something wrong. Immediately after diagnosis, parents struggled to make sense of their child's condition, often conducting their own research. Parents tended to focus on the physical consequences of stroke, which contributed to delays in identifying impacts to cognition in some instances. Identifying the stroke's cause was a key concern for many parents. Its absence sometimes fuelled guilt or blame towards self, ‘Is it your [my] fault that this happened?’,^74^ and in one instance towards healthcare professionals.^76^ While parents needed information, some felt overwhelmed by it,^74^ illustrating varying needs. Most studies reported inadequate responses from healthcare professionals. Communication was often perceived as blunt, jargon‐heavy, or rushed, which heightened distress. The acute care phase also left some parents, especially fathers, feeling sidelined and powerless: ‘It was the most helpless feeling’.^65^ Mothers often focused on the child's immediate needs, while fathers described broader family responsibilities, reflecting gendered strategies. This construct reveals the different methods used by parents to mobilize beyond shock, in addition to ways in which these attempts can be hindered.

#### Impact to parents, families, and the affected child: distress and sadness

A pervasive theme was continuing worry, particularly about recurrence, death, or future impairments: ‘I'm always afraid (…)’.^75^ This uncertainty left some parents in a heightened state of vigilance. One study described this experience as a ‘paralyzing fear of the unknown’,^65^ which was echoed across studies and was emphasized by another study titled ‘Grappling with uncertainty’.^74^ Emotional impacts included depressive symptoms and frequent tearfulness, especially earlier on, with one parent describing his partner becoming emotional ‘more hourly than weekly’.^74^ As a result of the emotional impact, family dynamics were noted to be negatively affected too. Only one study explored the experience of siblings in detail or noted positive changes in spousal relationships because of a child's stroke; as one father explained, ‘It's made it stronger. Much stronger’,^65^ about his relationship with his wife. There were limited but significant references to the reciprocal effects between parents' and children's emotional states, ‘… I was giving her all my anxiety, and therefore, she was becoming anxious …’,^72^ suggesting the child's well‐being was closely linked to that of the parent. This construct illustrates the profound impacts to parents' mental health and family dynamics.

#### Learning to manage, with a demand on resources

After discharge, many parents felt abandoned by hospital services and underprepared for care at home. Community agencies, including schools, were sometimes found to be helpful. Although some parents welcomed returning home, this was often overshadowed by the reality of complex care needs. Parents described upskilling themselves through online research, engaging with physical therapy, and advocating to navigate systems and support their child. This could be made more difficult by the timing of the child's stroke as one parent shared difficulties organizing support over the school holidays.^73^ One study added that parents initially spent up to 4 hours looking up information on the Internet and other sources every day, which reduced over time; one participant shared later recognition of a continuous cycle of learning, while another described the upskilling process as stressful but gratifying because it expanded the definition of her role as a parent.^76^ Although this illustrates a positive progression for parents beyond discharge, it is borne out of necessity: ‘We are the experts because there are no others’.^76^ Mothers were primarily responsible for this adaptation. The emotional and financial burden was substantial wherein caregiving was described as a ‘heavy load’. This was particularly highlighted in the study with low‐income families whose children were more severely affected, in which caregiving became all‐consuming: ‘… my life lives according to him […], I live for him like, totally’.^66^ It was also the only study that did not report mothers reducing employment, probably reflecting economic necessity. This illustrates the process parents, predominantly mothers, go through to find better ways to manage their child's condition. It comes with a great demand on resources that further deepens the emotional impact and distress felt by parents.

#### Socio‐spiritual resources

Support came from family, friends, peers, and faith, although these were not consistently discussed across studies. Faith‐based support appeared in only two studies^65^, ^66^ where it provided strength: ‘I believe God gave him to me because He knows that I am capable of taking care’.^66^ Although considered separately during analysis, these factors were consistently described as offering similar forms of support, primarily comfort, justifying their grouping under the term socio‐spiritual resources, as seen in similar findings.^77^ Peer support was largely positive, offering connection and hope, although some parents were not ready to engage.^74^ This illustrates the range of support sought by parents, which helped mitigate emotional strain, although not universally accessed or effective.

#### Living with and beyond stroke: adjusting and post‐stroke growth

Most studies described evolving parental perspectives, often marked by greater self‐awareness and acceptance, and less pessimism over time. One study^65^ highlighted a shift in fathers' priorities towards living in the present. Emotional support was desired, although only one study explored its effects,^65^ noting some fathers' reluctance to seek help. Adjustment remained a continuing process, especially for parents of more severely affected children. While some described adapting so fully that stroke faded into the background, ‘… we live with it … we don't even realize it at a certain point …’,^72^ others noticed cycles of continuous re‐adjustment as new challenges arose. This construct indicates that some adjustment occurs over time for parents, but that this can be influenced by various factors, including parental role, and that adjustment can be seen as a continuing process as children age and encounter more complex environments.

#### Normative ideals, expectations, and comparisons

Striving for normality was an emergent theme across studies. The emotional impact of deviation from normative expectations was profound for parents; one study was titled ‘Seeking normality …’^73^ while another highlighted the emotional toll of further marginalization and unmet ideals.^66^ Parents frequently questioned what ‘normal’ meant for their child and themselves, often exacerbating anxiety and complicating parental decisions, such as the choice between promoting independence or offering additional support. Some parents found relief in downward comparisons or reframing their expectations,^65^ creating a ‘new normal’ that included a mix of sadness, joy, and resilience: ‘Part of the new normal is disappointments, sadness … you'll be depressed some days; you'll be excited others …’.^65^ While societal norms remained influential, parents gradually adapted their ideals to better align with their lived realities.

### Overview of an explanatory model of parents' experiences

**FIGURE 1:**
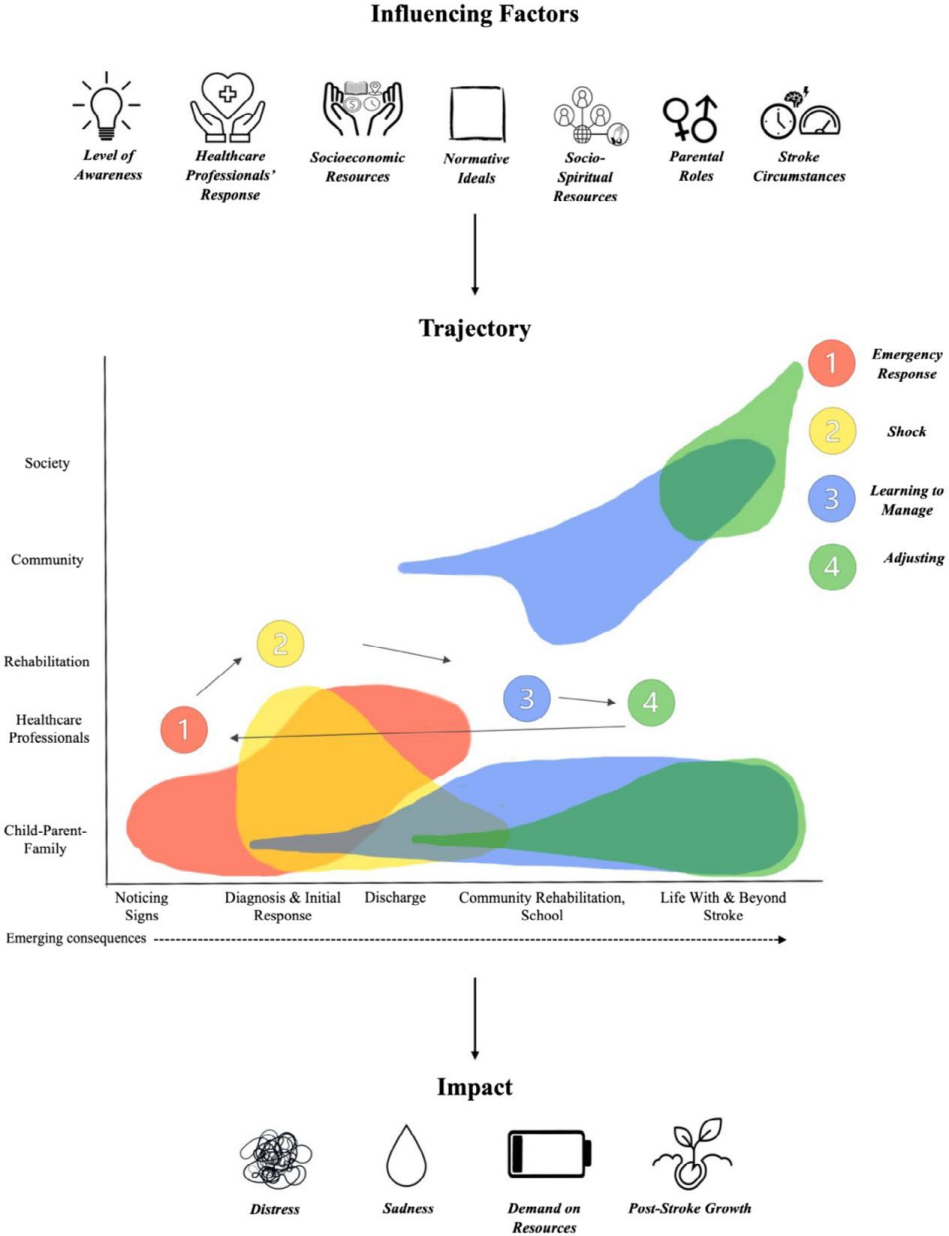
Proposed model of parents' experiences having a child who has had a stroke.

Key elements of parents' experiences as synthesized from the studies included in the meta‐ethnography are proposed in the model (Figure 1) consisting of three parts: influencing factors to parents' experiences; the trajectory of their experiences; and the impact of these experiences. Icons were used to enhance clarity and reflect study findings (e.g. male and female signs to represent maternal and paternal roles as these were the only ones referenced). Influencing factors included level of awareness, healthcare professionals' responses, socioeconomic resources, normative ideals, socio‐spiritual resources, parental roles, and stroke circumstances. The trajectory reflects the different stages of experiences and perspectives following stroke. This is mapped over time and across multiple levels of society generally in line with Bronfenbrenner's ecological systems model^18^ and the Lifespan Navigation‐Building Framework,^17^ but emphasizing parents' lived experiences over policy contexts. It moves from: (1) emergency response to (2) shock; (3) learning to manage; and finally (4) adjusting. Moving through the trajectory involves impacts to the child–parent–family, broadly captured as distress, sadness, a demand on resources, and post‐stroke growth. Given the evolving nature of developmental conditions, parents may cycle through the stages of the trajectory as new challenges emerge over time.

## DISCUSSION

This meta‐ethnography synthesized seven qualitative studies exploring parents' experiences of having a child who had a stroke. All studies, including an unpublished thesis, largely met acceptable quality standards. From these, third‐order constructs were developed, forming an explanatory model encompassing influencing factors, experience trajectory, and impacts. Notably, awareness of childhood stroke and support from healthcare professionals were identified as lacking, exacerbating parents' emotional impact, a demand on resources, and post‐stroke growth. Normative ideals were also found to play a key role in influencing parents' emotional responses.

Despite variations in circumstance, a common trajectory emerged across studies, beginning with emergency response and shock, followed by learning to manage, and eventually acknowledging and adjusting. This progression aligns with broader literature on parenting children with long‐term health conditions, which also highlights difficulties with healthcare support.^14^ However, unlike previous research highlighting continuing tensions as parents' growing expertise was undervalued by professionals, our findings indicate support faded over time. Similar patterns of unmet needs after discharge, especially for information and professional support, are noted in the literature on adult stroke,^78^ and from the perspective of parents with children who have disabilities.^79^, ^80^ Readiness for discharge is also linked to better outcomes for children and parents.^81^

While disbelief is reported in other childhood conditions (e.g. paediatric cancer and congenital heart disease^82^, ^83^), the profound lack of awareness of childhood stroke seems distinct. Regarding stigma, one study highlighted how teachers dismissed parents' concerns about this, possibly because of differing perspectives based on models of disability (medical model vs social model).^21^ This may reflect a broader shift as parents become more confident advocates, although changes in disability‐related views were not deeply explored. Wider research confirms stigma and oppression are common for families affected by childhood disability,^84^ and parents may internalize societal blame, aligning with minority stress theory.^85^ Nonetheless, the experiences of socioeconomically disadvantaged parents remain underexplored.^86^

Although socioeconomic context was not explicitly mentioned in included studies, context clearly shaped experiences when comparing across studies. This is supported by longitudinal data showing mothers in lower socioeconomic positions report worse health when caring for a disabled child.^87^ In such contexts, social and spiritual resources have been shown to alleviate distress.^66^, ^88^ Socio‐spiritual approaches, shown to benefit patients in palliative care^77^ and those with cancer,^89^ also hold promise in childhood disability.^90^, ^91^ Social support, particularly peer support, has proved helpful to parents whose children have learning disabilities or chronic disability,^92^, ^93^ although quantitative evidence of its efficacy remains mixed.

The findings revealed under‐researched areas that reflect broader systemic patterns in child health research. There were notable gaps in attention to faith, ethnicity, culture, sex, diverse family structures, and consideration of siblings and the wider family unit. Spiritual needs are often overlooked in paediatric settings, limiting holistic care.^94^ The minimal representation of those from ethnic minority backgrounds in increasingly diverse societies, and evidence of poorer health outcomes for these groups, confronts us with the need for more inclusive research.^95^ Similarly, fathers' perspectives and the voices of parents from more diverse and broader family structures remain largely absent from paediatric research,^96^, ^97^ which calls for a more critical stance on caregiving norms and institutional practices. Further, despite evidence that siblings' well‐being is closely tied to family functioning and long‐term adjustment following childhood chronic illness,^98^ considerations of their experiences in current research were mostly absent. Positive experiences were also limited in the findings. Despite quantitative evidence that parents want more psychological support, this was only mentioned in two studies. These omissions may reflect cultural biases in research priorities rather than an absence of such experiences in families' lives, which have implications for equitable and inclusive care practices. Future research should carefully consider ways to incorporate these aspects, such as through purposive sampling and approaches that adopt cultural humility, to ensure a richer and more representative understanding of parents' experiences.

The results offer insights across timepoints, parental roles, and socioeconomic backgrounds, although the last two aspects were only considered in a few studies alongside aforementioned underrepresented areas. This limits the transferability of findings to more diverse family and social contexts. This review included studies from a range of countries with varied focuses including stroke‐related circumstances, and most studies were conducted in the past decade, adding to the model's relevance. Input from advisory group members added depth and emotional texture to the meta‐ethnography process through sharing their own personal experiences. Additionally, their involvement supported unique but prominent aspects of parents' experiences to be more clearly identified and highlighted the assumptions of RB, encouraging closer inspection of the studies to ensure a robust and representative analysis. Consistency across studies further supports the proposed explanatory model in terms of coherence and adequacy; however, one study^76^ lacked reports of ethical approval and anonymity, which may have affected the trustworthiness of its findings. One study was not peer‐reviewed, which calls into question its level of scrutiny and verification of findings. The inclusion of the study by Ramos et al.,^66^ in which the age at having a stroke was ambiguous, deviates from a strict paediatric definition and may have affected the transferability of these results as some of the participants in the study might not have had their condition through childhood. This study also introduced a potential language bias because it was translated to English from the original in Portuguese.

## CONCLUSIONS

Parents' experiences of childhood stroke follow a trajectory similar to other paediatric conditions, yet the suddenness and severity of stroke, and the widespread unawareness that it can affect children, make their experiences uniquely distressing. This highlights a critical need for research, awareness campaigns, and clinical guidance specific to childhood stroke. Parents require sustained and tailored support from healthcare professionals, not only in the acute phase but also throughout their post‐stroke journeys into long‐term adjustment. This includes helping to navigate healthcare systems, developing care skills, and managing continuing uncertainty. Healthcare professionals should be trained to deliver information in a compassionate, paced, and responsive manner that considers the whole family's context and their unique needs, such that parents can be supported with their self‐directed learning at a pace that suits them. Practitioner training should also incorporate awareness of systemic inequities and support for less‐considered family members, including fathers and siblings, and culturally and structurally diverse families. Embedding psycho‐education and emotional support into routine care may help buffer distress while encouraging parents' confidence and autonomy in managing their child's needs as well. Equitable access to social, economic, and socio‐spiritual resources is also essential, particularly for families facing structural disadvantage. Societal perceptions of disability, shaped by ableism, continue to burden parents, demonstrating the need for systemic change to promote more inclusive and supportive environments. Although the biopsychosocial model has shifted thinking around disability, it has arguably continued to centre biological aspects. Reframing it as a socio‐psycho‐biological model^99^ could prompt greater recognition of the social and psychological dimensions of families' experiences and foster more holistic care. Finally, the model proposed through this synthesis offers a foundation for future research. Researchers could explore its utility through longitudinal studies or use it to develop and evaluate interventions tailored to different stages of the parental trajectory. Testing the model's applicability across conditions and cultures will also be key to improving its transferability and impact.

## CONFLICT OF INTEREST STATEMENT

Payment was offered to one author who was involved as an advisory group member, however monetary compensation was not a motivating factor for her contributions to this research.

## Supporting information


**Figure S1:** Preferred Reporting Items for Systematic Reviews and Meta‐Analyses (PRISMA) flow diagram generated by Covidence.

**Table S1:** Example of stages for a reciprocal analysis.

**Table S2:** Confidence in the Evidence from Reviews of Qualitative Research (CERQUAL) responses for synthesis findings.

**Table S3:** Summaries of contextual information from the studies selected for the review.

**Table S4:** Results of the critical appraisal.

**Table S5:** Results for the critical appraisal with details of identified strengths and weakness.

## Data Availability

The data supporting this systematic review and meta‐ethnography consist of previously published articles, which are publicly available in the academic literature. Extracted data and analytic materials generated during this study are available from the corresponding author upon reasonable request.
