# Supplementary figures and images for: Parents' experiences of having a child who had a stroke: A systematic review and meta‐ethnography
Source: Dev Med Child Neurol. 2025 Sep 26;68(2):187–98. doi: 10.1111/dmcn.70004 (PMC12766557; doi:10.1111/dmcn.70004)

**Figure S1:** PRISMA flow diagram generated by Covidence<sup>38</sup>

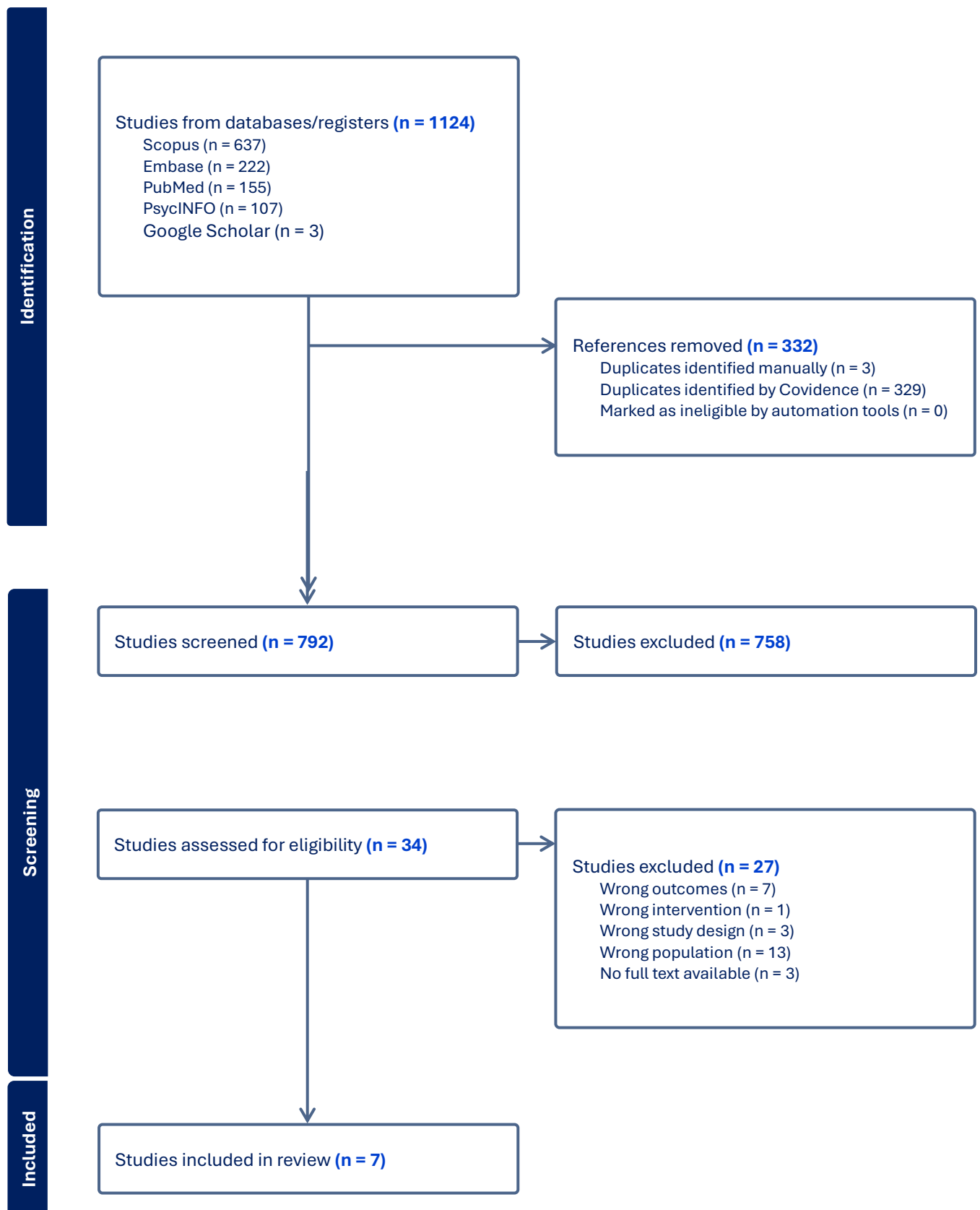

Supplement: Supplementary file 1 — Figure S1: Preferred Reporting Items for Systematic Reviews and Meta‐Analyses (PRISMA) flow diagram generated by Covidence. [file DMCN-68-187-s005.pdf]
