# Supplementary material for: Parents' experiences of having a child who had a stroke: A systematic review and meta‐ethnography
Source: Dev Med Child Neurol. 2025 Sep 26;68(2):187–98. doi: 10.1111/dmcn.70004 (PMC12766557; doi:10.1111/dmcn.70004)
Supplement: Supplementary file 2 — Table S1: Example of stages for a reciprocal analysis. [file DMCN-68-187-s003.docx]

Table S1 Example of stages for a reciprocal analysis

| Order & author | Initial themes | 1st order constructs | 2nd order constructs | Translations | Synthesis - reciprocal |
| --- | --- | --- | --- | --- | --- |
| 1. McKevitt | (2nd guilt for possible family as cause) | - | A minority referred to feelings of guilt because of potential connections between the stroke and family genetic conditions, or a more general concern that the stroke had occurred because of something a parent did or did not do. | Minority expressed guilt/blame - genetic or action/inaction | Guilt, blame, responsibility  McKevitt et al shares that only a minority expressed feelings of guilt or blame attributing themselves (genetically) responsible for their child's injury.  Khan et al. also reported parents' sense of guilt, blame, and parental responsibility for their child's injury, suggesting this is induced to fill in a void of explanation. |
| 3. Khan et al. | (1st&2nd blame & guilt)  (1st&2nd uncertainty --> taking responsibility & focus on cause) | You think about, like, and I mean you do beat yourself up emotionally, and I was like, ‘Is it your [my] fault that this happened? (Sheila, mother of James)   “Is this why he’s like this?” There was a lot of that | Parents, especially mothers, expressed feelings of guilt and personal blame, even when they were aware the stroke was not their fault  Given the uncertainty about the exact aetiology of the stroke in many cases, there remains a void to be filled with an explanation, to the extent that parents cause themselves to believe that they are responsible for the event. | Guilt, personal blame & responsibility (void to be filled) |  |

*Note*. Key for colours: guilt/blame, focus on cause, lack of information
