# Supplementary material for: Parents' experiences of having a child who had a stroke: A systematic review and meta‐ethnography
Source: Dev Med Child Neurol. 2025 Sep 26;68(2):187–98. doi: 10.1111/dmcn.70004 (PMC12766557; doi:10.1111/dmcn.70004)
Supplement: Supplementary file 3 — Table S2: Confidence in the Evidence from Reviews of Qualitative Research (CERQUAL) responses for synthesis findings. [file DMCN-68-187-s002.docx]

Table S2 CERQual responses for synthesis findings

|  | Methodological limitations | Coherence | Adequacy | Relevance | CERQual assessment of confidence in the evidence |
| --- | --- | --- | --- | --- | --- |
| Shock of an Inexplicable Diagnosis | Moderate: researcher reflexivity lacking across studies except an unpublished thesis, limited recruitment pool for 2 studies, no mention of ethical approval in 1 study, 2 studies provided minimal quotes | No or very minor concerns: 1 study only describes shock in relation to another condition | Minor concerns: clear across all but 1 study | Moderate concerns: Range of countries represented, but only one from the Global South, range of stroke severities including some with comorbidities. Less traditional parenting set ups were not represented | Moderate confidence |
| Emergency Response and Experiences of HCPs | Moderate: researcher reflexivity lacking across studies except an unpublished thesis, limited recruitment pool for 2 studies, no mention of ethical approval in 1 study, 2 studies provided minimal quotes | Minor concerns: 1 study does not describe parents’ responses in earlier phases | Minor concerns: clear across all but 1 study | Moderate concerns: Range of countries represented, but only one from the Global South, range of stroke severities including some with comorbidities. Less traditional parenting set ups were not represented | Moderate confidence |
| Impact to Parents, Families, and the Affected Child | Moderate: researcher reflexivity lacking across studies except an unpublished thesis, limited recruitment pool for 2 studies, no mention of ethical approval in 1 study, 2 studies provided minimal quotes | No or very minor concerns | No or very minor concerns | Moderate concerns: Range of countries represented, but only one from the Global South, range of stroke severities including some with comorbidities. Less traditional parenting set ups were not represented | Moderate confidence |
| Learning to Manage, with a Demand on Resources | Moderate: researcher reflexivity lacking across studies except an unpublished thesis, limited recruitment pool for 2 studies, no mention of ethical approval in 1 study, 2 studies provided minimal quotes | Minor concerns: 1 study did not mention this aspect as its focus was on earlier stages | Minor concerns: clear across all but 1 study | Moderate concerns: Range of countries represented, but only one from the Global South, range of stroke severities including some with comorbidities. Less traditional parenting set ups were not represented | Moderate confidence |
| Socio-Spiritual Resources | Moderate: researcher reflexivity lacking across studies except an unpublished thesis, limited recruitment pool for 2 studies, no mention of ethical approval in 1 study, 2 studies provided minimal quotes | Moderate concerns: only briefly mentioned in most studies, 2 studies emphasise this aspect in more detail | Moderate concerns: only briefly mentioned in most studies, 2 studies emphasise this aspect in more detail | Moderate concerns: Range of countries represented, but only one from the Global South, range of stroke severities including some with comorbidities. Less traditional parenting set ups were not represented | Low confidence |
| Normative Ideals, Expectations, and Comparisons | Moderate: researcher reflexivity lacking across studies except an unpublished thesis, limited recruitment pool for 2 studies, no mention of ethical approval in 1 study, 2 studies provided minimal quotes | No or very minor concerns | No or very minor concerns | Moderate concerns: Range of countries represented, but only one from the Global South, range of stroke severities including some with comorbidities. Less traditional parenting set ups were not represented | Moderate confidence |
| Living With and Beyond stroke: Adjusting | Moderate: researcher reflexivity lacking across studies except an unpublished thesis, limited recruitment pool for 2 studies, no mention of ethical approval in 1 study, 2 studies provided minimal quotes | Minor concerns: 1 study did not mention this aspect, and only briefly described in 1 study | Minor concerns: 1 study did not mention this aspect, and only briefly described in 1 study | Moderate concerns: Range of countries represented, but only one from the Global South, range of stroke severities including some with comorbidities. Less traditional parenting set ups were not represented | Moderate confidence |
