# Supplementary material for: Parents' experiences of having a child who had a stroke: A systematic review and meta‐ethnography
Source: Dev Med Child Neurol. 2025 Sep 26;68(2):187–98. doi: 10.1111/dmcn.70004 (PMC12766557; doi:10.1111/dmcn.70004)
Supplement: Supplementary file 4 — Table S3: Summaries of contextual information from the studies selected for the review. [file DMCN-68-187-s004.docx]

Table S3 Summaries of contextual information from the studies selected for the review

| Author, Year of Publication, Location | Aims | Participant Selection Methods | Study Design, Data Collection, and Analysis | Sample Details |
| --- | --- | --- | --- | --- |
| Leal Martins et al., 2021, Switzerland | Quantitative: 1) To examine the timeline of care from early reported symptoms to formal diagnoses confirmed through imaging for neonates who had neonatal arterial ischaemic stroke (NAIS) Qualitative: 2) To explore parents' experiences of the timeline of events and their perspectives related to delays in diagnosis, prognosis discussion, and current view on their child’s quality of life 3) To identify areas for improvement | Inclusion criteria: Families whose children were treated in the Geneva University Hospitals and met the neonatal arterial ischaemic stroke (NAIS) diagnostic criteria, excluding premature or other cerebrovascular injuries Quantitative:  Sampling method: Retrospective sampling (2008-2017) Recruitment method: Data were collected from the Swiss Neuropediatric Stroke Registry and a locally established database Qualitative:  Sampling method: Convenience sampling Recruitment method: Participation was offered to families in-person in the hospital's outpatient child neurology clinic, excluding those who could not speak in French | Methodology: Mixed methods research; quantitative component using newborns' and mothers’ medical records, and qualitative component using questions developed with research, clinical expertise, and informal discussions with PPE, focused on event chronology, diagnosis announcement, and quality of life for standardised, open-ended interviews between 2018-2019 Analysis: a timeline of care and estimated time to diagnosis was identified, followed by thematic content analysis of qualitative interviews | Quantitative: 14 neonates (12 males, 2 females) Qualitative: 12 French-speaking parents (9 mothers, 3 fathers) to 9 children who had a NAIS 2 to 10 years prior to interview resulting in 1 child with slight and 2 children mild motor deficits, 3 children with attention deficits and 2 children with ongoing seizures |
| Khan et al., 2022, the Northeast of England | To explore early parental experiences of having a child who had a perinatal stroke, including the impact to parents' psychological wellbeing | Inclusion criteria: Parents whose children were term or pre-term and predominately had a stroke on one side of their brain (as identified through neuroimaging) identified within the first 3 months of their life Sampling method: Convenience sampling of participants already involved in a feasibility trial between 2015-2017 Recruitment method: Participation was offered in-person, using flyers codesigned with PPE | Methodology: Qualitative research using a topic guide developed with PPE, focused on parents' psychological wellbeing and the need for related support for in-depth interviews with both parents where possible Analysis: Integrated qualitative analysis drawing from grounded theory^100^, analytic induction^101^, and constructionist grounded theory | 16 parents (6 interviews with just mothers, 5 with both parents) to 11 children who had a perinatal stroke resulting in 5 children with no/mild deficits and 7 children with moderate/severe deficits; children were 5 to 6 months corrected gestational age at the time of interview |
| McKevitt et al., 2019, England | To report the experiences of families whose child had a stroke from their own, and not a clinical, perspective, including perceptions of quality of care and support strategies used | Inclusion criteria: Parents whose children had a stroke Sampling method: Purposive sampling of parents based on age at stroke and time since stroke Recruitment method: Recruited from a sample of participants from 3 regional specialist services who previously completed a needs survey | Methodology: Qualitative research using a topic guide developed with PPE for semi-structured interviews Analysis: Thematic analysis^102^ | 12 parents to children who had a paediatric stroke <1-year to <5-years prior to interview |
| Soufi et al., 2017, the Rhone-Alpes region, France | 1) To document the experiences of parents whose child had experienced a stroke 2) To understand parents’ awareness of stroke-related behavioural and cognitive consequences (hypothesising that this was underestimated in the early stages of rehabilitation) | Inclusion criteria: Families whose children were aged between 1 month and 17 years with an ischaemic or haemorrhagic arterial stroke within the past 1-5 years, excluding those with perinatal stroke or unrelated cognitive/behavioural disorders Sampling method: Purposive sampling of families based on sociodemographic factors (i.e., age, occupation, education level, etc.) from databases of the Saint-Etienne and Lyon hospitals and rehabilitation clinics Recruitment method: Participation was initially offered by mail | Methodology: Qualitative research using a guide focused on the stroke event and parents' related feelings, the child's functioning pre-stroke, experiences returning home and to school, managing changes to life, and future outlook for a mix of individual and couple-based semi-structured interviews  Analysis: Data content analysis | 14 parents (13 mothers (mean age = 35.4), majority graduated from high school at the least, and few were unemployed) including one adoptive mother, 4 fathers (mean age = 39) to children who had a stroke between 0.8 to 4.8 years prior to interview |
| Grover, 2014, USA | To understand parents' self-directed learning experiences having a child who had a stroke | Inclusion criteria: Parents whose children had a paediatric stroke within the past 6 years Sampling method: Purposive sampling to identify parents who directed their own learning to understand the implication of stroke Recruitment method: Recruited through advertisement on the Children's Hemiplegia & Stroke Association (CHASA) website | Methodology: Qualitative research using open-ended interviews, conducted during an annual CHASA retreat Analysis: Qualitative data analysis^103^ | 7 mothers (mean age = 37, all married, majority Caucasian (n=5), all but one had at least a bachelor's degree, 4 unemployed) to children who had a paediatric stroke at the age of 25 days to 5 years |
| Ramos et al., 2020, the Zona da Mata Mineira, Brazil | To illuminate the experiences of mothers of children who had a stroke because of sickle cell disease (SCD) | Inclusion criteria: Mothers whose children were aged 6 to 29 years who had a stroke due to SCD Sampling method: Snowball sampling through initial referral of one participant by the president of the Association of People with Sickle Disease | Methodology: Qualitative research using^83^ existential phenomenology focused on participants' lives with their children and the meaning they ascribed to this for phenomenological interviews Analysis: Qualitative data analysis^104^ | 10 mothers (aged 25 to 48 years, with all but one having an income between 1-2 minimum wages, half had not completed elementary school and only one had completed high school) to children aged 10-24 years who had a stroke due to SCD (6 had comorbidities) |
| Robbins, 2014 (thesis; not peer reviewed), Philadelphia, USA | To share the stories of fathers whose child had a stroke, exploring the challenges they have experienced and the strategies they have found helpful and healing | Inclusion criteria: Fathers whose children were aged between 9 months and 18 years with mild to moderate deficits due to stroke or the family has been informed of the possibility of emerging deficits Sampling method: Purposive sampling of fathers (biological or acting in role) living with their child and the child's mother based on race, SES, child's gender, time since stroke, stroke deficit and impact severity, and religious affiliation Recruitment method: Recruited through their child's participation in a paediatric stroke program at the Children's Hospital of Philadelphia in clinic or via flyers | Methodology: Qualitative research using a guide derived from grounded theory^105^ for in-depth semi-structured interviews Analysis: Grounded theory^105^ | 13 fathers (aged 31-50 years), all married with 2-3 children over half had the lowest academic achievement of only completing high school, only one was unemployed, nearly half had no/minimal financial burdens. The most common religions were Christian denominations (n=9), and fathers’ ethnicities were Caucasian (n=9) or African American (n = 4) to children who had a stroke 9 months to over four years prior to interview, 8 had seizures following stroke and 4 had another significant diagnosis beyond stroke |

*Note. Presented in order initially read, determined by chronology and relevance*

1. Charmaz K. Constructing grounded theory: a practical guide through qualitative analysis. In: Introducing Qualitative Methods. Thousand Oaks; 2006. p. 223.
2. Seale C. The quality of qualitative research. *Qualitative Inquiry*. 1999; **5**(4):465-78.
3. Braun V, and Clarke V. Using thematic analysis in psychology. *Qual Res Psychol*. 2006; **3**(2):77–101.
4. Creswell JW, Creswell JW. Qualitative inquiry and research design: choosing among five approaches. 3rd ed. Los Angeles: SAGE Publications; 2013. p. 448.
5. Heidegger, Martin: Beiträge zur Philosophie (Vom Ereignis) [Internet]. 2003 [cited 2025 Mar 31]. Available from: https://www.klostermann.de/Heidegger-Beitraege-zPhilos3A-Ln
6. Corbin JM, Strauss A. Grounded theory research: procedures, canons, and evaluative criteria. *Qual Sociol*. 1990; **13**(1):3–21.
