# Supplementary material for: Parents' experiences of having a child who had a stroke: A systematic review and meta‐ethnography
Source: Dev Med Child Neurol. 2025 Sep 26;68(2):187–98. doi: 10.1111/dmcn.70004 (PMC12766557; doi:10.1111/dmcn.70004)
Supplement: Supplementary file 5 — Table S4: Results of the critical appraisal. [file DMCN-68-187-s001.docx]

Table S4 Results of the critical appraisal

| CASP Criteria | Leal Martins et al., 2021 | Khan et al., 2022 | McKevitt et al., 2019 | Soufi et al., 2017 | Grover, 2014 | Ramos et al., 2020 | Robbins, 2014 |
| --- | --- | --- | --- | --- | --- | --- | --- |
| Was there a clear statement of the aims of the research? | Yes | Yes | Yes | Yes | Yes | Yes | Yes |
| Is a qualitative methodology appropriate? | Yes | Yes | Yes | Yes | Yes | Yes | Yes |
| Was the research design appropriate to address the aims of the research? | Can’t tell | Yes | Yes | Yes | Yes | Yes | Yes |
| Was the recruitment strategy appropriate to the aims of the research? | Yes | Can’t tell | Yes | Yes | Can’t tell | Yes | Yes |
| Was the data collected in a way that addressed the research issue? | Yes | Yes | Yes | Yes | Can’t tell | Yes | Yes |
| Has the relationship between researcher and participants been adequately considered? | Yes | Can’t tell | Can’t tell | Yes | Can’t tell | Can’t tell | Yes |
| Have ethical issues been taken into consideration? | Yes | Yes | Yes | Yes | No | Yes | Yes |
| Was the data analysis sufficiently rigorous? | Can’t tell | Yes | Can’t tell | Can’t tell | Can’t tell | Yes | Yes |
| Is there a clear statement of findings? | No | Yes | Yes | Yes | Yes | Yes | Yes |
| Numerical score | 7 | 8 | 8 | 8.5 | 6.5 | 8.5 | 9 |

*Note.* Numerical score was calculated assigning ‘Yes’ a value of 1, ‘Can’t tell’ a value of 0.5, and ‘No’ a value of 0.
