# Supplementary material for: Parents' experiences of having a child who had a stroke: A systematic review and meta‐ethnography
Source: Dev Med Child Neurol. 2025 Sep 26;68(2):187–98. doi: 10.1111/dmcn.70004 (PMC12766557; doi:10.1111/dmcn.70004)
Supplement: Supplementary file 6 — Table S5: Results for the critical appraisal with details of identified strengths and weakness. [file DMCN-68-187-s006.docx]

Table S5 Results for the critical appraisal with details of identified strengths and weakness

| ***Example of Stages for a Reciprocal Analysis*** CASP Criteria | Leal Martins et al., 2021 | Khan et al., 2022 | McKevitt et al., 2019 | Soufi et al., 2019 | Grover, 2014 | Ramos, 2020 | Robbins, 2014 |
| --- | --- | --- | --- | --- | --- | --- | --- |
| Was there a clear statement of the aims of the research? | Response: Yes Strengths: Clearly defined objectives with a justified rationale Weaknesses: Less clarity on how each aim would be explored | Response: Yes Strengths: Clear and simply worded aim Weaknesses: Aims were not explicitly labelled | Response: Yes Strengths: Clear aims including justified rationale and a defined target audience Weaknesses: Ambiguity about how data from another source would be integrated to inform service provision | Response: Yes Strengths: Succinct and well-structured aims, including a justified sub-focus with a clear hypothesis Weaknesses: The rationale for excluding perinatal stroke was unclear | Response: Yes Strengths: Simple and clear aim Weaknesses: Aims were not explicitly justified or labelled | Response: Yes Strengths: Simple and concise objective, clearly labelled and well justified Weaknesses: Objectives did not incorporate purpose or intended outcomes | Response: Yes Strengths: well justified specific aims and research questions outlined including intended audience Weaknesses: Aims were difficult to find as information was not clearly labelled and was available in different parts |
| Is a qualitative methodology appropriate? | Response: Yes  Strengths: Qualitative approach (as part of mixed-methods study) to explore aspects of aims was contextualised and seems appropriate | Response: Yes Strengths: Qualitative approach seems appropriate in line with aims | Response: Yes  Strengths: Qualitative approach seems relevant to aims | Response: Yes  Strengths: Qualitative approach deemed appropriate to aims | Response: Yes  Strengths: Qualitative approach seems appropriate in line with aim | Response: Yes Strengths: Qualitative research within a justified theoretical framework seems in line with aims | Response: Yes Strengths: qualitative approach clearly justified. Use of grounded theory as framework explained and seen as suitable. |
| Was the research design appropriate to address the aims of the research? | Response: Can’t tell Strengths: Qualitative interviews seem fitting with aims | Response: Yes Strengths: Qualitative interviews seem appropriate in accordance with aims | Response: Yes Strengths: The use of semi-structured interviews seems well considered | Response: Yes Strengths: Semi-structured interviews seem appropriate in line with aims | Response: Yes Strengths: Semi-structured, open-ended interviews seem fitting with aims | Response: Yes Strengths: Qualitative interviews seem fitting in line with aims. | Response: Yes Strengths: Use of semi-structured, in-depth interviews seen as appropriate |
| Was the recruitment strategy appropriate to the aims of the research? | Response: Yes  Strengths: Clear, relevant, and justified inclusion and exclusion criteria in addition to recruitment procedures Weaknesses: No detail provided for how the sample size was determined | Response: Can’t tell Strengths: Recruitment materials developed with PPE and inclusion criteria seemed partly fitting  Weaknesses: Limited recruitment pool misaligned with general aim with no justification of including non-primary caregivers | Response: Yes Strengths: Recruitment across regions and age of stroke onset fits with broader aim Weaknesses: Participants limited to those who completed previous research and could not be sampled by broader demographic factors | Response: Yes Strengths: Appropriate inclusion criteria in line with aims, clearly detailed with consideration for socio-economic factors, and data saturation reached Weaknesses: No explicit details of sampling choices | Response: Can’t tell Strengths: Appropriate approach to identify relevant participants, highlighted by clear inclusion criteria Weaknesses: Ambiguity regarding how participants were selected, participants were only chosen within a hemiplegia and stroke charity, and no explicit justification for stroke onset criterion | Response: Yes Strengths: Clear approach seen as appropriate. Recruitment through an established association with equitable aims Weaknesses: No justification for age of child criterion | Response: Yes Strengths: Inclusion criteria relevant to aims Weaknesses: Participants only recruited from a specific stroke program |
| Was the data collected in a way that addressed the research issue? | Response: Yes Strengths: Data collection methods, procedure, and setting clearly outlined with relevant topic guide comprehensively developed Weaknesses: No justification for use of standardised interviews and no direct involvement of PPE | Response: Yes Strengths: Topic guide used with consideration to areas of importance according to PPE Weaknesses: No provision of interview guide or detail of modifications mad, and no mention of data saturation | Response: Yes Strengths: Topic guide developed with PPE Weaknesses: No mention of how sample size was determined and limited information about how the interview guide was developed, and the potential effect of varied settings was not highlighted | Response: Yes Strengths: Parents had autonomy over format (alone or together), field notes conducted, transparency in interviewers’ relationship with participants, modifications made as result of initial theme generation.  Weaknesses: No mention of influence of field notes/interviewer on data | Can’t tell Strengths: Interviews conducted at a time of increased salience Weaknesses: Ambiguity in interview process as no mention of topic guide or data saturation | Response: Yes Strengths: Brief and open questions that fit with aims, and a field diary was reportedly used Weaknesses: No mention of how field diary was incorporated or whether data saturation was met | Yes Strengths: use of interview guide, field notes, and post-interview interviewer reflections Weaknesses: No provision of interview guide |
| Has the relationship between researcher and participants been adequately considered? | Response: Yes Strengths: Reasoning provided for interviewer choice and adjustments made to questionnaire following additional input from relevant stakeholders. Weaknesses: No reported reflexivity | Response: Can’t tell Strengths: Interviews conducted in participants’ homes, indicating some consideration of creating a comfortable interview environment Weaknesses: No reported consideration of relationship between participants and interviewers or researchers and data or reflexivity | Response: Can’t tell Strengths: Choice of impartial interviewer justified Weaknesses: Lack of reflexivity | Response: Yes Strengths: Report of researcher backgrounds, motivations and interviewer choice Weaknesses: Lack of reflexivity or consideration for factors that may have influenced data collection/interpretation | Can’t tell Strengths: Member checking used, which indicates researchers valuing participant input Weaknesses: No mention of relationship or reflexivity | Response: Can’t tell Strength: A clear intention to accurately encompass each individual within the analysis Weaknesses: No mention of relationship or reflexivity | Response: Yes Strengths: Reflexivity woven throughout study; potential conflicts of interests highlighted and exclusion criteria adjusted accordingly; role of interviewer considered in detail in relation to participants and attempts to address it made Weaknesses: No standardised protocol used for reflections or acknowledgement of potential influences in results |
| Have ethical issues been taken into consideration? | Response: Yes Strengths: Informed consent obtained and approval by ethics committee with decisions made to increase participant autonomy Weaknesses: No further consideration reported for participants’ wellbeing | Response: Yes Strengths: Consent obtained, and ethical approval received; pseudonyms used; interviews taken part in participants’ homes, which potentially aids with power imbalances.  Weaknesses: No reported consideration for participant wellbeing or mention of debrief practices | Response: Yes Strengths: NHS ethics received, and the interview setting was determined by parents, indicating respect for their choices Weaknesses: No reported consideration for study’s impact on participants | Response: Yes Strengths: ethical approval received; consent obtained; choices offered to participants; anonymised transcripts.  Weaknesses: No mention of consideration of impact on participants | Response: No Strengths: Informed consent of participants was obtained Weaknesses: Ethical approval not explicitly reported; no briefing/debriefing mentioned; names used with no mention of pseudonyms or preservation of anonymity | Response: Yes Strengths: Ethical approval received on numerous levels; participants consented; data was presented anonymously Weaknesses: No mention of participant debrief practices | Yes Strengths: Ethics approval received on numerous platforms; power differentials acknowledged and redressed; clear care for the potential emotionality of interviews given with plans in place if additional support was needed; details of monetary reimbursement provided Weaknesses: Participants could only withdraw their data by sending the author a letter |
| Was the data analysis sufficiently rigorous? | Response: Can’t tell Strengths: Conveyed relevant information succinctly supported with participant quotes Weaknesses: No explanation for using thematic content analysis or details of analytic process; minimal inclusion of contradictory data | Response: Yes Strengths: Details of analytic process provided included robust presentation of findings Weaknesses: Lack of reported consideration of interviewer, interview guide, or motivations/backgrounds of researcher team leaves interpretations without context | Response: Can’t tell Strengths: Rich thematic descriptions provided alongside the frequency of similar experiences reported by participants; some inclusion of contradictory data Weaknesses: Limited quotes to support analysis provided | Response: Can’t tell Strengths: Explanation of steps involved in analysing data; evidence of contradictory data; information succinctly presented and checked by participants.  Weaknesses: Choice of analysis seems less relevant for aims; brief supporting quotations provided; no explanation for why some participants did not review themes or changes made as a result of consulting with participants | Response: Can’t tell Strengths: Peer debriefing and review with participants adds robustness to analysis; comprehensive analysis with plenty of quotes from participants with context Weaknesses: No detail of analysis provided; results section lengthy with numerous concepts repeated. | Response: Yes Strengths: Clearly laid out themes Weaknesses: Slightly confusing use of language (may be due to translation) | Response: Yes Strengths: Details of analysis clearly provided, including multiple rounds of coding and team discussion of differences; some contradictory data provided; transparency with initial plans vs reality provided Weaknesses: Results were repetitive in sections with lots of long quotes provided |
| Is there a clear statement of findings? | Response: No Strengths: Clearly listed main themes Weaknesses: Themes largely matched standardised questionnaire; less attention paid to qualitative data in discussion section | Response: Yes Strengths: Comprehensive but succinct summary; outlines potential improvements; integrates findings into existing models | Response: Yes Strengths: Comprehensive summary of findings in line with aims that were also generalised to other findings | Response: Yes Strengths: Clear summary provided with a conceptual framework; findings expanded to other literature | Response: Yes Strengths: Summary broadly encompasses findings; easy to read, fitting with potential intended audience (parents) | Response: Yes Strengths: Summary broadly encompasses findings | Response: Yes Strengths: Clear summary provided |
| Numerical score | 7 | 8 | 8 | 8.5 | 6.5 | 8.5 | 9 |
